# Special Issue: Emerging Technologies for Medical Imaging Diagnostics, Monitoring and Therapy of Cancers

**DOI:** 10.3390/jcm10061327

**Published:** 2021-03-23

**Authors:** Mohsen Beheshti, Felix M. Mottaghy

**Affiliations:** 1 Division of Molecular Imaging and Theranostics, University Hospital, Paracelsus Medical University, 5020 Salzburg, Austria; 2 Department of Nuclear Medicine, University Hospital, RWTH University, 52074 Aachen, Germany; fmottaghy@ukaachen.de; 3 Department of Radiology and Nuclear Medicine, Maastricht University Medical Center, 6229 Maastricht, The Netherlands

Molecular imaging and therapy play an increasingly important role in the field of “precision medicine” as an emergent prospect for management of the cancerous disease. In this novel approach, individual variabilities such as the specific molecular biomarkers, genes, environment, and lifestyle of each person will be considered. This is, however, different from our current treatment concept, in which the prevention, diagnostic and therapy strategies are developed for a general population, with less focus on the distinctness between individuals. Our routine clinical practice of medicine was based on “personalized medicine” for decades, tailoring treatments to each patient. However, “precision medicine” considers the diagnostic and treatment approaches that are more precisely customized to specific molecular targets. In this era, the theranostic approach with targeted radionuclide therapy has unique promise in the precise management of cancers, as both the targeted vehicle and the radionuclide can be tailored to the individual patient.

The present Special Issue in the *Journal of Clinical Medicine* aims to focus on novel advancements and future trends of molecular imaging and therapy as well as the theranostic approach in the primary assessment and monitoring of disease in various cancers. In addition, this issue will also highlight the very important aspect of the use of artificial intelligence in the feature analysis of the molecular images, in relation with molecular biomarkers and their potential roles in “precision medicine”.

Several interesting topics are derived from the body of this scientific collection. In alignment with most advanced theranostic targets, Sankaranarayanan et al. [1] presented the recent pre-clinical advancements in imaging and theranostic developments of nuclear DNA repair enzyme Poly-(ADPribose) polymerase 1 (PARP1)-targeted radio-theranostics. The potential of different PARP-labelled radiotracers for imaging, therapy and disease-monitoring of various cancers has been discussed in this article. Among them, 18F-FTT and 18F-PARPi have been translated to clinical trials, showing promising results in ovarian and head–neck cancers, respectively. The authors reported that, upon reproducible validation in the future, 18F-Olaparib could also be a potential candidate for clinical trials due to its favorable chemical characteristics. The future trends of the nano-formulated PARPis have also been discussed in this review article. In another review article, Yordanova et al. [2] showed the promising new agents in the molecular imaging and therapy of neuroendocrine tumors (NETs), novel combination therapies and new applications of existing molecular imaging modalities in nuclear medicine. In addition to somatostatin receptors, the authors discussed the current developments, focusing on novel theranostic targets such as C-X-C motif chemokine receptor 4 (CXCR4), fibroblast activation protein (FAP) and glucagon-like peptide-1 (GLP-1) receptors in molecular imaging and therapy of NETs, as well as non-invasive characterization of tumor biology.

Cancer immunotherapy using immune-checkpoint inhibitors (ICI) has revolutionized the therapeutic landscape of various malignancies like non-small-cell lung cancer or melanoma and has opened a new era of precision medicine. However, pre-therapy response prediction and assessment during ICI treatment is challenging due to the lack of reliable biomarkers and the possibility of atypical radiological response patterns. By reviewing the current data, Lang et al. [3] showed the promising impact of biomarkers derived from PET/CT for the development of personalized treatment strategies, especially concerning the choice of therapy, longitudinal management of the disease and prognostic aspects.

In alignment with the individualized treatment approaches, Manafi-Farid et al. [4] provided an overview of bone-seeking radiopharmaceuticals used for bone-pain palliation, and their effectiveness and toxicity, as well as the results of the combination with other therapies. In the setting of personalized medicine, the authors proposed an algorithm for the selection of an appropriate radiopharmaceutical for bone-pain palliation therapy.

In a retrospective study, Li et al. [5] assessed the potential of 18F-FDG PET/MRI versus MRI alone in the primary staging and restaging of 34 patients with rectal cancer. They showed that 18F-FDG PET did not yield a significant improvement in diagnostic accuracy of PET/MR in staging of rectal cancer, since MR alone facilitated the accurate classification of disease stage, with good-to-excellent inter-observer agreement. However, in a study with a similar design in 51 rectal cancer patients, Manafi-Farid et al. [6] showed that 18F-FDG PET/CT revealed influential information for more accurate staging in 12.9% of patients, and more importantly, led to a management change in 24.1%, mainly in the determining of the radiation field or dose. They concluded that 18F-FDG PET/CT is not expected to replace MRI. However, the invaluable potential role of 18F-FDG PET/CT in the management of anal carcinoma may advocate for its routine use, along with pelvic MRI, in the clinical practice.

In line with the diagnostic potentials of PET/CT imaging, Paymani et al. [7] evaluated the diagnostic performance of 18F-Fluorocholine and 68Ga-PSMA PET/CT in prostate cancer, two potential radiotracers that have been presented in recent years. The results of this study showed a superior detection rate of 68Ga-PSMA PET/CT, especially in the early detection of metastases in patients with biochemical recurrence and low PSA levels of <1.0 ng/mL. However, they assumed that in selected hormone-resistant high-risk prostate cancer patients, 18F-Fluorocholine PET/CT may improve overall diagnostic accuracy.

In two clinical studies, Urbano et al. [8,9] correlated 99m-Tc-Sestamibi SPECT findings with histopathological bio-characteristics in parathyroid adenoma and breast cancer. They showed breast osteoblast-like cells as potential markers for breast-specific gamma imaging with 99m-Tc-Sestamibi for the detection of breast cancer lesions. They also reported that data obtained on patients with positive or negative 99m-Tc-Sestamibi scintigraphy support the hypothesis that sestamibi can be a tracer that is capable of predicting some biological characteristics of parathyroid tumors, such as angiogenesis, proliferation indexes, and the invasion of surrounding tissues or vessels.

In accordance with the latter research, Lee et al. [10] showed that the analysis of imaging features using a combined imaging–histology grade provides more specific, practical results for survival prognosis in oral squamous cell carcinoma of the mandible.

With the exponential development and integration of artificial intelligence (AI) and deep learning (DL) that provide promising new aspects for improving patient care, four articles in this Special Issue are focused on these subjects.

Leitner et al. [11] examined the performance of multiparametric MRI-based radiomics in conjunction with AI for the assessment of breast cancer receptor status and molecular subtypes. They indicated that radiomics signatures derived from multiparametric MRI enable the determination of certain treatment-naïve molecular breast cancer subtypes with high accuracy. In addition, multiparametric radiomics imaging biomarkers could potentially serve as auxiliary parameters and a non-invasive method to derive prognostic and predictive information from the entire tumor before and during treatment. In line with the latter subject, Mahmood et al. [12] presented the promising results of a multistage mitotic-cell-detection technique of a faster-region convolutional neural network (Faster R-CNN) and deep CNNs for the assessment of breast cancer histopathology images. The strengths of this work comprise the adoption of Resnet-50 for the feature extraction in the Faster R-CNN, extraction of appropriate features in the post-processing, and score-level fusion of the Resnet-50 and Densenet-201 classifiers.

In another study, Arsalan et al. [13] evaluated two multiclass chest-X-ray segmentation networks to segment lung, heart, and clavicle bones to aid medical specialists in the diagnosis of cardiomegaly and other related diseases. The authors claimed that this novel method provides a fine segmentation performance in non-ideal scenarios and multiclass fashions, which may play an important role assisting with clinical and other automated interpretation and disease classification methods based on chest X-ray.

Finally, in a preliminary study by Takagi et al. [14], the authors examined whether deep learning can be applied to the analysis of cerebral hemodynamics and brain activity based on functional near-infrared spectroscopy (fNIRS) data. They found about a 90% accurate identification rate using convolutional neural network by measuring the changes in the concentration of cerebral oxygenated hemoglobin (oxy-Hb) and deoxygenated hemoglobin (deoxy-Hb). The findings are promising and emphasize the impact of deep learning for the evaluation of brain activity in different physiologic and pathologic conditions.

Our Special Issue comprises comprehensive preclinical and clinical data focusing on the impact of molecular imaging, therapy and theranostic approaches in the management of various cancers. Furthermore, the translation of artificial intelligence and deep learning into the clinical setting has been discussed in different articles. In conclusion, this Special Issue highlights part of the ongoing scientific efforts in the field of “precision medicine”.
